# The role, regulation and application of plant fruit trichomes

**DOI:** 10.1186/s43897-025-00167-x

**Published:** 2025-08-08

**Authors:** Ying Fu, Meng Li, Wei Zhang, Xueting Liu, Li Huang, Sen Zhang, Xinyue Liang, Liuxin Zhang, Kexuan Tang, Jocelyn K. C. Rose, Qian Shen

**Affiliations:** 1 Plant Biotechnology Research Center, Fudan-SJTU-Nottingham Plant Biotechnology R&D Center, School of Agriculture and Biology, Shanghai Jiao Tong University, Minhang, Shanghai 200240 China; 2 Plant Biology Section, School of Integrative Plant Science, Cornell University, Ithaca, NY 14853 USA; 3 School of Design, Shanghai Jiao Tong University, Minhang, Shanghai 200240 China; 4 Present Address: Key Laboratory of Plant Design, CAS Center for Excellence in Molecular Plant Sciences, Institute of Plant Physiology and Ecology, Chinese Academy of Sciences, Shanghai, China; 5 Present Address: Yazhouwan National Laboratory, Sanya, China

**Keywords:** Fruit trichome, Transcription factor, Post-harvest, Water loss

## Abstract

Trichomes, hair-like specialized epidermal structures on the surface of most plant organs, play key roles in plant defense against herbivores, reducing water loss, and shielding plants from UV radiation, among other functions. Controlling trichome development and the biosynthesis of trichome-derived specialized metabolites is a common defensive strategy adopted by plants to protect themselves from environmental stresses. However, trichomes exhibit distinctive functions in different plant tissues. Fruits, being the most economically valuable organs of many horticultural plants, often have trichomes on their surface. Nevertheless, there is a notable lack of research on the regulation and function of fruit trichomes, in comparison to the extensive studies conducted on trichomes in other plant tissues. Further investigation is needed to elucidate the specific functions of fruit trichomes. The regulation of plant trichome development and the multiple roles of trichomes represent a dynamic area of plant biology with significant implications for agriculture and biotechnology. This review aims to enhance the understanding of the functions, regulatory mechanisms, and applications of fruit trichomes, emphasizing their importance in advancing agricultural sustainability and productivity.

## Introduction

Most plant species have hair-like epidermal structures in their seed coat and aerial parts. Trichomes, originating from epidermal cells, are known to provide protection against various environmental and biotic stresses, including ultraviolet radiation, water retention, extreme temperatures, heavy metal absorption, and pathogen and insect attacks (Kundan et al. 2019; Wang et al. 2021b). Moreover, trichomes are excellent models for studying cell fate, cell differentiation, cell polarity, and cell expansion (Pattanaik et al. 2014; Wu et al. 2023a; Yang et al. 2013). The morphology of trichomes varies significantly among plant species, ranging in size and shape, and can be composed of single or multiple cells (Hülskamp 2004; Wagner et al. 2004). They may be glandular or non-glandular, and branched or unbranched (Kabir et al. 2024). In some plant species, the trichomes undergo further specialization, such as fruit spines in cucumber, the fruit prickles in *Rosa roxburghii* (Huang et al. 2022), and prickles on stem in eggplant or plants of the *Rose* genus (Zhang et al. 2021a; Zhong et al. 2021). Despite their disparate morphologies, trichomes on stems, leaves, seeds, and other parts serve similar protective functions across various plant species. Non-glandular trichomes play an important role in responding to extreme environments and preventing herbivorous insect movement and feeding (Han et al. 2022). Glandular trichomes, on the other hand, serve as storage and synthesis compartments for specialized plant metabolites, many of which are used in food additives, pharmaceuticals, flavors, and natural pesticides (Feng et al. 2021).

However, the specific functions of trichomes in particular regions or tissues remain largely unstudied. As research has progressed, it has become evident that trichome functions are more complex than previously understood. For example, cotton petal trichomes act as natural Velcro, playing a crucial role in maintaining the shape of the flower buds and ensuring the production of seeds (Tan et al. 2016). Similarly, the interlocking trichomes at the anther margin are necessary for forming a closed anther cone in tomato flower, anchoring the edges of neighboring anthers and facilitating self-pollination. These anther cone trichomes play a pivotal role in enabling the tomato plant to complete the process of self-pollination (Wu et al. 2024a).

Fruits are the most valuable organs of many horticultural crops. However, the study of fruit trichomes is relatively underexplored compared to the abundant research on trichomes in other plant parts. The fruit surface significantly influences external appearance and post-harvest shelf life (Berhin et al. 2022). This disparity indicates the need for further research to gain a comprehensive understanding of the regulatory mechanisms and functions of fruit trichomes. This review synthesizes current knowledge on the molecular regulation of fruit trichome development and explores their functions, with a particular focus on post-harvest water loss and potential applications for improving shelf life.

### The functions and regulation of fruit trichome development in Tomato

Tomato (*Solanum lycopersicum*), one of the most economically important vegetable crops in the world, is a well-established model for studying fruit development and trichome biology. In tomatoes, there are seven different types of trichomes, each with distinct morphologies and functions (Simmons et al. 2005). Unlike the unicellular trichome of *Arabidopsis*, all tomato trichomes are multicellular. Types II, III, and V are non-glandular and composed of neck and base cells, while types I, IV, VI, and VII are glandular, featuring a glandular cap capable of storing and secreting various secondary metabolites (Chalvin et al. 2020). Tomato is the most intensively studied horticultural plant regarding the regulation of trichome initiation and development. However, almost all studies have focused on leaf and stem trichomes. For a long time, tomato fruit trichomes were not considered in studies of mature fruit since they are largely absent after harvest. Nevertheless, these fruit trichomes are abundant when the fruit remains undisturbed (Ying et al. 2020).

### The functions of tomato fruit trichomes

Tomato fruit transpiration directly influences fruit freshness and taste. There are no stomata on the surface of tomato fruit, and the water loss occurs mainly through cuticle transpiration. The cuticle is mainly composed of a lipid matrix cutin intertwined with a polysaccharide fraction derived from the epidermal cell wall (Nawrath 2006). Its hydrophobic nature allows the cuticle to protect the plant against water loss and regulate water exchange. Consequently, the thickness and composition of the cuticle have long been considered critical to the final post-harvest quality of tomatoes (Fernandez-Munoz et al. 2022; Lara et al. 2019). However, a study reported that after screening a tomato core collection of 398 accessions, the transpirational water loss did not correlate with various measures of cuticle abundance or composition. Rather, there was a strong dependence on the abundance of polar pores on the tomato surface (Fich et al. 2020). These pores were associated with fruit trichomes and were exposed when fruit trichomes were broken. Tomato fruit trichomes were readily broken during handling, harvesting, and storage. In addition, in contrast to the rapid healing of the stem scar, broken fruit trichome had limited self-sealing of the exposed pores (Fich et al. 2020).

The relationship between fruit trichome density and post-harvest water loss provides a new perspective and technological route for improving fruit shelf life and explains previously reported unusual phenomena. Due to the reduction of cutin synthesis, leaf water conductance and chlorophyll leaching were increased in *pe* (a new allele of Cutin Deficient 2, CD2) leaves compared to AC plants (Nadakuduti et al. 2012). Interestingly, cutin levels in fruit of tomato *cd2* and *cd3* mutants were reduced by more than 90% compared to the parental M82 line, but *cd2* and *cd3* had a minimal effect on postharvest water loss (Isaacson et al. 2009). Similarly, *shn2* fruit also showed a dramatic reduction in cutin of the fruit cuticle. However, compared to control fruit, postharvest fruit water loss was not increased but greatly reduced in the *shn2* mutant (Bres et al. 2022). We believe that the unusual post-harvest water loss in fruit described above may have overlooked the regulatory role of fruit trichome density in water loss.

### Genes involved in the formation of tomato fruit trichomes

**Fig. 1:**
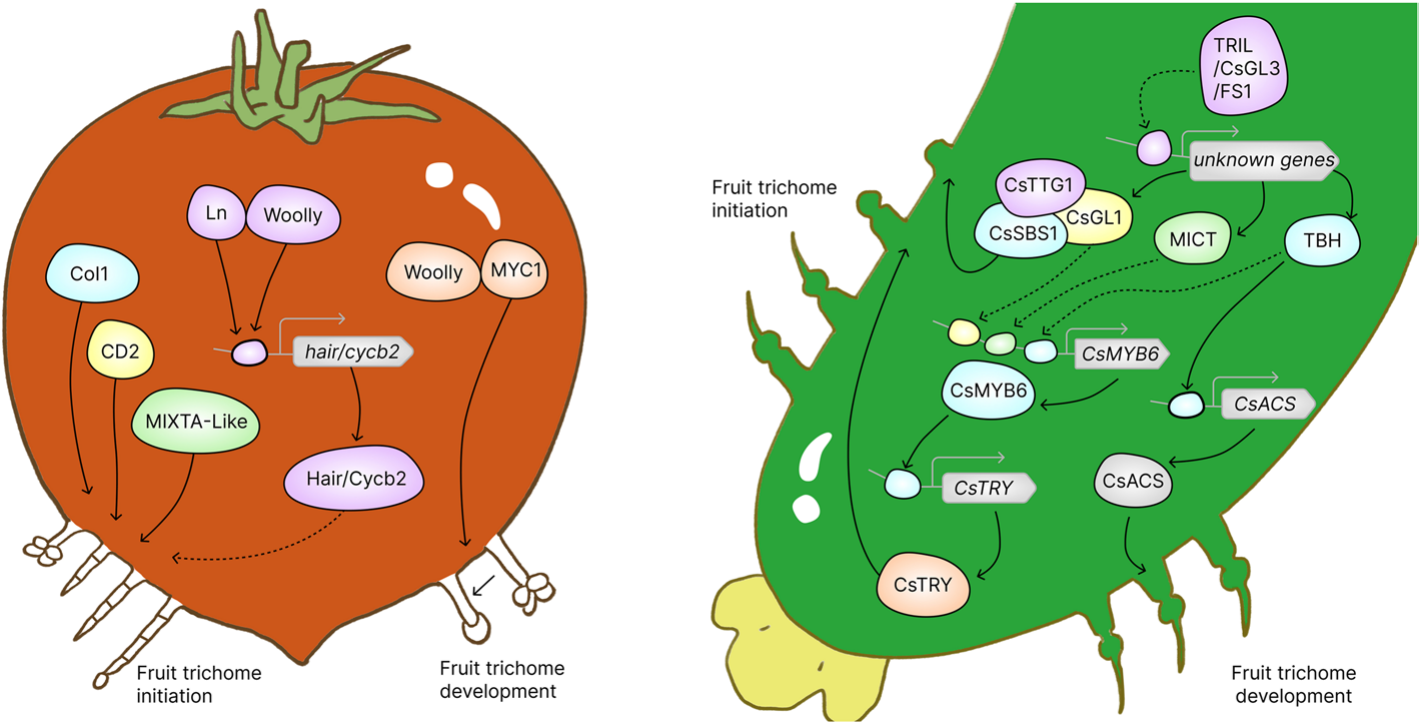
The regulatory models of fruit trichome initiation and development in tomato and cucumber. Dotted line, indirect interaction; continuous line, direct interaction

Most studies investigating leaf and stem trichome initiation and development have not conducted phenotypic observations in fruit trichomes, currently it is unclear whether all these genes known to be involved in leaf or stem trichomes affect fruit trichome formation. Compared to other tissues of tomato plant, the functions and molecular mechanisms underlying fruit trichome development were poorly understood. Only a few regulators involved in tomato fruit trichome initiation were identified (Fig. 1). Most studies have only described the phenotypic characteristics of fruit trichomes, lacking research on the corresponding regulatory mechanisms. For example, CORONATINE-INSENSITIVE1 (COI1), an F-box protein that was required for JA signaling, played an important role in regulating tomato fruit glandular trichomes. The loss-of-function mutation in *COI1* exhibited a complete absence of fruit trichomes, giving the tomato fruit a smooth and glabrous appearance (Li et al. 2004). In addition, the *Lanata* (*Ln*) mutant in Micro-Tom showed excess trichomes in stem, leaves and fruits, indicating that *Ln* controls trichome formation in tomato fruits (Vendemiatti et al. 2017). Additionally, SlMIXTA-like, a metabolic regulator gene identified by mGWAS analysis, overexpressed under the fruit-specific *E8* promoter enhanced trichome formation in tomato fruit (Ying et al. 2020). The *CD2* gene, which encodes a member of HD-ZIP IV family, was associated with a fruit-specific reduction in cuticle biosynthesis (Isaacson et al. 2009). The *cd2* mutant displayed several phenotypes, including a significant reduction in fruit trichome density (Nadakuduti et al. 2012).

### The functions and regulation of fruit trichome development in cucumber

Cucumber (*Cucumis sativus* L.) was one of the most important vegetable crops cultivated worldwide (Huang et al. 2009). The cucumber fruit was covered with trichomes (also known as spines), which were highly specialized structures derived from epidermal cells (Yang et al. 2018). Cucumber fruit trichomes were among the most studied plants and served as a model for fruit trichome research. There were two types of trichomes have been identified in cucumber fruits, both of which were multicellular (Chen et al. 2014). Type I trichomes (bloom trichomes) had a glandular structure and produced white powdery secretions composed primarily of silicon dioxide. Type I trichomes were responsible for the rough outer appearance of the fruit. Type II trichomes (fruit spines, the dominant type in cucumber) were larger, with a sharp spiny stalk and a conical or spherical base (Chen et al. 2014).

### The functions of cucumber fruit trichomes

Unlike *Arabidopsis thaliana* trichomes, cucumber fruit trichomes offered both research models and practical economic value. The density and size of cucumber fruit trichomes were key fruit quality traits that influenced fruit appearance and market value (Chen et al. 2014; Li et al. 2015). For example, fruits with large spine base were preferred in China fresh market cucumber, while smooth fruits were more popular in European cucumbers (Yang et al. 2019). Smooth cucumbers were easier to clean, pack, transport, and store (Yang et al. 2014). Additionally, smooth fruit had lower pesticide residues and a more pleasant taste. The smooth cucumber fruit gained popularity in regions that traditionally preferred large spine base cucumbers due to its attractive and shiny appearance (Cui et al. 2016; Yang et al. 2019). Although no research currently examined the impact of cucumber fruit trichomes on postharvest quality, they were of significant value. Thus, understanding the regulatory mechanisms underlying the cucumber fruit trichome formation was of great interest for enhancing breeding programs and the economic value of cucumber production.

### Genes and phytohormones involved in the formation of cucumber fruit trichomes

Cucumber fruit spines were regulated by a complex gene network involving multiple transcription factors. To understand fruit trichome development in cucumber, trichome-related mutant cucumbers were important to determine the key regulators for their initiation and formation. Several mutants, such as *trichome-less (tril)* (Wang et al. 2016), *glabrous3 (csgl3)* (Cui et al. 2016; Pan et al. 2015), *tiny branched hair (tbh)* (Chen et al. 2014), *micro-trichome (mict)* (Zhao et al. 2015b), *glabrous1 (csgl1)* (Li et al. 2015), and *few spines 1 (fs1)* (Zhang et al. 2016) disrupt fruit trichome development through various mechanisms. The *glabrous 3* (*csgl3*) and *trichome-less* (*tril*) mutants showed a completely glabrous phenotype on fruit epidermis (Cui et al. 2016; Wang et al. 2016; Zhao et al. 2015a) (Fig. 1). Interestingly, the *csgl3*, *tril*, and *fs1* mutants were allelic, caused by different mutations in the *Csa6G514870* gene, which encoded a class IV homeodomain-leucine zipper (HD-Zip IV) transcription factor involved in cucumber trichome initiation (Du et al. 2020). The other three mutants (*csgl1*, *tbh* and *mict*) lacked visible trichomes on fruit surfaces but possessed many extremely small trichomes with stunted morphology (Chen et al. 2014; Li et al. 2015; Zhao et al. 2015b). Thus, trichome density remained unaffected, suggesting that *CsGL1/TBH*/*Mict* were involved in trichome development rather than initiation (Li et al. 2015). It was speculated that Tril and CsGL3 may directly bound to *CsTBH*, *MICT*, and *CsGL1* to regulate multicellular fruit trichome development.

In addition, several genes regulating spine initiation and development were identified. HD- ZIPs, MYBs transcription factors, and WD-repeat proteins regulated trichome fate determination and initiation in cucumber fruit. *CsTTG1*, a WD40 repeat containing gene, played an important role in regulating fruit spine formation through direct protein–protein interaction with CsGL1 (Chen et al. 2016). Overexpression of *CsMYB6*, encoding a MIXTA-like MYB transcription factor, reduced the density of spines in cucumber by directly binding to the promoter region of *CsTRY* (Yang et al. 2018). CsTBH was involved in multicellular trichome development by binding to the promoters of *CsACS* (Zhang et al. 2021b) (Fig. 1). A novel regulator network was proposed that the CsGL1/CsSBS1/CsTTG1 complex significantly regulated spine formation and size (Yang et al. 2022b).

Phytohormones such as cytokinin (CK), auxin (IAA), and gibberellin (GA) modulated cucumber fruit trichomes development. *CsTu* was involved in CK biosynthesis by indirectly promoting the expression of two *CHL* genes, stimulating fruit trichomes initiation process (Yang et al. 2014). CsHEC2 promoted wart formation by acting as an important cofactor for CsGL3 and CsTu to directly stimulating CK biosynthesis in cucumber (Wang et al. 2021c). *CsTS1* was induced by auxin treatments, and regulated fruit trichomes development through increasing auxin levels in cucumber (Yang et al. 2019). *CsGA20ox1* negatively regulated fruit spine development by modulating the GA signaling pathway (Li et al. 2015). CsTTG1 acted as regulatory proteins involved in mediating the control of fruit spine development by key genes involved in cell wall loosening and phytohormone GA (Guo et al. 2020).

### The functions and regulation of fruit trichome development in peach

Peach (*Prunus persica*) was one of the most economically important fruit crops worldwide. The presence of pubescence, also called fruit trichomes, on the surface of peaches characterized them as fuzzy fruits. Like cucumber spines, the peach fruit trichomes were important agronomic traits that served as the basis for the variety identification and contributed to their market value. Peach fruit trichomes were non-glandular and unicellular. Similar to *Arabidopsis* trichomes and cotton fibers, these trichomes were deeply rooted in the epidermis with a thin lumen and thick cell walls (Fernández et al. 2011).

### The functions of peach fruit trichomes

**Fig. 2:**
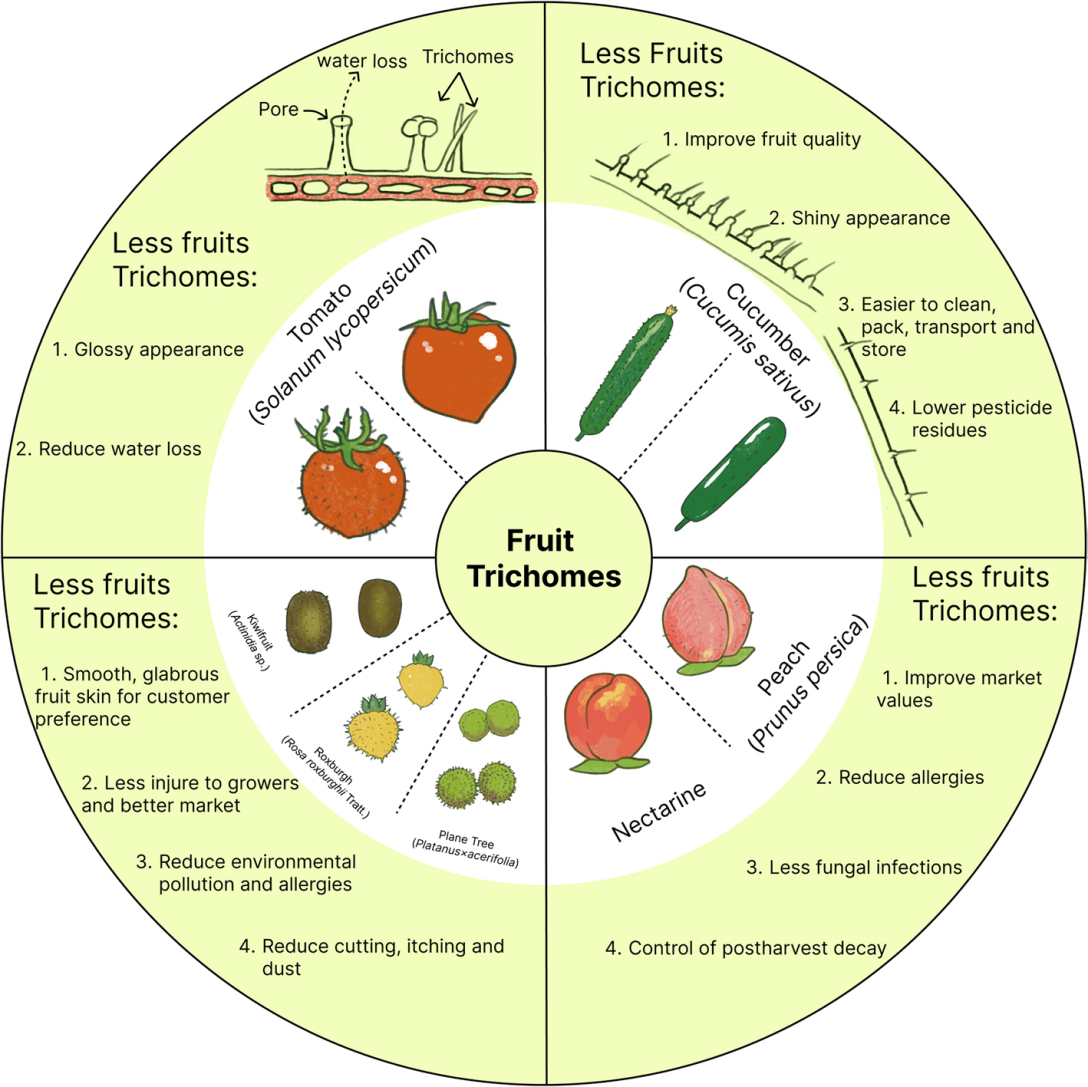
Fruit trichomes in tomato, cucumber, peach, kiwifruit, Roxburgh, and plane tress. Fruit trichomes impact various aspects of fruit maintenance, including fruit water loss, fruit quality, appearance, production process, customer preference, environmental pollution, allergies, microorganism invasion, and postharvest decay

Peach fruit remained pubescent throughout the growing season, the presence or absence of skin pubescence was one of the commercial characteristics used to classify peach fruits. Like other plant trichomes, the trichome on the peach epidermis was considered to be a natural physical barrier with multiple biological functions, such as disease defense, transpiration regulation, and UV protection (Wang et al. 2021b). However, in commercial peach production, the fruit trichomes were often brushed off prior to marketing, immediately after harvest (Siddiq et al. 2012). This raised the question of why peach trichomes were removed despite their beneficial functions. One potential explanation may be related to allergies. The allergens Pru p 1 and Pru p 3 were identified as primary contributors to peach-related allergies, and the Pru p 3 protein was localized within the trichomes of ripe peaches (Cubells-Baeza et al. 2017). Nectarines were widely cultivated and played an important role in world peach production. In addition to their glossy or shiny skin, nectarines had decreased allergenic properties compared to fuzzy peaches, the Pru p 3 protein was undetectable in the nectarine (Botton et al. 2006). Another possible rationale for peaches being shaved was their association with brown rot and fruit surface microbiome. Peach fruit had a short shelf life due to serious fungal infections during postharvest storage. In China, the incidence of brown rot caused by *Monillia spp*. was much higher than that of other postharvest diseases (Luo et al. 2022). After peach trichomes were removed, the incidence of postharvest brown rot decreased significantly, and bacterial diversity declined, suggesting that trichome removal was beneficial for the control of postharvest decay (Shen et al. 2023). It was important to note that the integrity of the epidermal layer of fruit surface was critical for fruit water retention. Although the rate of postharvest water loss in peach fruit is higher in shaved peach fruit than in intact fruit, it was important to note that the integrity of the fruit surface may have been compromised by the remove off the fruit trichomes, similar to what occurred with the rubbing of tomato trichomes (Fig. 2). Taken together, we proposed that breeding peach varieties with fewer or trichome-free fruits would not only reduce the risk of allergies, but also affect the microbial communities on the fruit surfaces and extend postharvest shelf life by reducing water loss.

### Genes involved in the formation of peach fruit trichomes

Although peach fruit trichomes were morphologically similar to *Arabidopsis* trichomes, only a few regulatory factors were identified as being involved in regulating the initiation and development of fruit trichomes in peach. Possible reasons for this were the shortage of peach mutants without fruit trichomes, difficulties in genetic transformation, and the long growth cycle of peaches, all of which limited further study of the genes that regulate peach fruit trichomes using genetic engineering techniques. As a subspecies of peach, the fruit trichome (pubescence) was absent on the skin of nectarine. The peach/nectarine (G/g) trait was controlled by a classical G locus previously mapped on chromosome 5, with the nectarine trait being recessive to peach fuzzy fruit (Dirlewanger et al. 2006). In combination with fine mapping and NGS-based variant discovery, the candidate gene for this G locus was identified as the *PpeMYB25* gene. An insertion of an LTR retroelement in exon 3 of the *PpeMYB25* gene was identified as the cause of the recessive glabrous phenotype (Vendramin et al. 2014). Another peach trichome regulator was identified as the R2R3 MYB transcription factor PpMYB26. Phylogenetic analysis showed that it was closely related to PpMYB25 and shared a conserved MIXTA motif (Yang et al. 2022a). The MIXTA motif of the R2R3 MYB family subgroup 9 normally controlled trichome formation, which has been studied in other plants (Scoville et al. 2011; Shi et al. 2018; Ying et al. 2020; Zhao et al. 2020). The PpEXPA4 was homologous gene to GhEXPA1 which promotes cotton fiber elongation (Shan et al. 2014). It was speculated that PpEXPA4 functioned downstream of PpMYB25 and PpMYB26, involving in the regulation of peach fruit trichome formation. However, whether it affected the initiation or elongation of peach fruit trichomes required further investigation. Spatial transcriptome sequencing technology, which provided ultra-high resolution of transcriptomic information, emerged as a powerful tool for research in the field of biological development. Based on spatial transcriptome sequencing, a novel trichome-related marker gene, *Prupe.7G196500*, was identified in peach. Gain-of-function analysis in Arabidopsis showed that *Prupe.7G196500* promotes trichome development (Liu et al. 2024; Zhao et al. 2023).

### The functions and regulation of fruit trichome development in other plants

Kiwifruit (*Actinidia* sp.) was another fruit species with a diversity of fruit trichome types, which affected its popularity in the commercial market. However, it remained largely unknown which genes mediate trichome development in kiwifruit. The surface trichomes of *A. deliciosa* ‘Hayward’ cultivar were generally considered a commercial disadvantage (Miao et al. 2023). In contrast, the *A. arguta* cultivar which had smooth, glabrous fruit skin, was often used in breeding programs to develop trichome-less skin for customer preference (Baranowska-Wójcik et al. 2019). In *A. latifolia*, another kiwifruit species with short and sparse fruit trichomes, alternative splicing of NAP1 was responsible for the shorter trichomes (Miao et al. 2023). The relationship between fruit trichomes and post-harvest water loss remained inconclusive due to conflicting findings. For example, two kiwifruit cultivars,'Xuxiang'(short shelf life) and'Hayward'(long shelf life), were subjected to scanning electron microscope observation and measurement. The results indicated that the fruit trichomes of'Xuxiang'were longer, thicker, and denser than those of'Hayward', which resulted in higher water loss of'Xuxiang'during post-harvest storage (Gao et al. 2020). Despite *A. arguta* has a typical trichome-less skin, it has a relatively short storage and shelf-life compared to the cultivar *A. deliciosa* ‘Hayward’ (Baranowska-Wójcik et al. 2019; Lu et al. 2024). Further investigation was required to clarify the relationship between kiwifruit fruit trichomes and water loss.

*Platanus acerifolia* (Aiton) Willd. was a commonly used landscape tree. Its fruit trichomes, which shed and dispersed in the air, causing environmental pollution and seriously affecting people's health and livelihoods (Zhang et al. 2019). Several genes from *P. acerifolia* have been identified as regulators of trichome development via heterologous expression in Arabidopsis. These include *PaMYB82* (Zhang et al. 2019), *PaTCP4* (Shao et al. 2021), *PaNAC089* (Shao et al. 2022), and *PaGL1-like* genes (Zhang et al. 2020). However, the molecular mechanisms required further exploration to develop glabrous varieties.

*Rosaceae* plants have prickles growing on the pericarp and stalks. These prickles, which were epidermal projections formed by multiple cellular divisions. A study indicated that prickles were modified glandular trichomes that continued to grow and eventually hardened into their final prickle morphologies (Kellogg et al. 2011). Compared to the fruit prickles with thin cell walls, stem prickles possessed thick cell walls contain fewer organelles and plastids (Wang et al. 2021a). Chestnut rose (*Rosa roxburghii* Tratt.) was a species of the *Rosaceae* family with high nutritional and medicinal value, had two main types of trichomes present on the fruit surface: flagelliform and acicular trichomes (Wang et al. 2021a, 2019). These trichomes, especially acicular trichomes, injure growers and negatively impact production, management, consumption, and marketing. A recent study showed that *RrTTG1* in *R. roxburghii,* a homolog of *TRANSPARENT TESTA GLABRA1* (*TTG1*) in *Arabidopsis*, may play a similar regulatory role in initiating trichome development (Huang et al. 2022).

### The applications of fruit trichomes

Fruit trichomes were diverse structures with multiple applications and implications for fruit quality, commercial value, and environmental adaptability (Fig. 2). Recent studies in tomatoes highlighted that water loss during postharvest storage was not solely determined by cuticle thickness or composition but was significantly influenced by the pores exposed due to fruit trichome breakage during handling and storage (Fich et al. 2020). Consequently, reducing trichome density on fruits surface was proposed as a strategy to minimize postharvest water loss and extend shelf life. This observation provided new insights into previous findings in mutants with drastically reduced cutin or cuticle levels, such as *cd2*, *cd3*, and *shn2*, which exhibited minimal or even reduced impacts on postharvest water loss (Bres et al. 2022; Isaacson et al. 2009). In other crops like cucumbers and kiwifruits, fruit trichome density substantially affected both consumer preferences and storage performance. Smooth-skinned fruits with few or reduced fruit trichomes offered advantages such as easier cleaning, transport, and reduced pesticide residues (Baranowska-Wójcik et al. 2019; Yang et al. 2014). Understanding the developmental regulation of fruit trichomes in these crops could enhance breeding programs and economic outcomes.

In peaches, fruit trichomes provided physical and biological protection but posed challenges, including allergenicity and increased susceptibility to microbial contamination (Shen et al. 2023). Studies have shown that removing fruit trichomes reduced allergenic properties and postharvest decay. However, trichome removal compromised the fruit epidermal integrity and increased water loss. These contrasting effects underscored the need for a balanced approach in trichome-related regulations, considering both their protective functions and the potential trade-offs in fruit quality and storage.

In addition, the regulatory mechanisms underlying fruit trichome development not only offered pathways to improve postharvest quality and market value but also advanced evolutionary and taxonomic studies (Arteaga et al. 2021; Serna et al. 2006). For example, traits and natural mutations associated with fruit trichomes in *Arabidopsis* natural populations were linked to evolutionary adaptations to climate and precipitation (Arteaga et al. 2021).

## Discussions

### Similarities and differences lie in plant trichome development across different tissues

Developmental differences between fruit trichomes and those on leaves or stems remained an underexplored area, with limited studies directly addressing this topic. In cucumbers, research primarily focused on fruit trichome development, with minimal attention given to trichomes on leaves or stems. Notably, some studies identified genes, such as *CsMict*, *CsTril*, and *CsMYB6*, that influenced trichome initiation not only on fruits but also on other organs, including flowers, leaves, and hypocotyls (Du et al. 2020; Pan et al. 2021; Zhao et al. 2020). However, despite this shared genetic regulation, the morphology and structure of cucumber fruit trichomes differed significantly from those on other tissues.

In contrast, in tomato, the focus of trichome research has largely been on leaves and stems, leaving fruit trichomes relatively understudied. Nevertheless, certain genes, including *MIXTA-like*, *CD2*, *MYC1*, and *WOOLLY*, which regulate leaf and stem trichome development, also demonstrated effects on fruit trichome initiation and growth, as evidenced by phenotypic photographs of the fruits (Hua et al. 2021; Nadakuduti et al. 2012; Wu et al. 2024b; Ying et al. 2020). These findings suggest that while fruit trichomes and those on leaves or stems may share common regulatory genes, their development may differ and may be influenced by tissue-specific spatial and temporal mechanisms. For example, in tomato, the *H* and *HL* genes, which regulate trichome initiation in leaves and stems, displayed spatial and temporal differences in their regulatory roles across tissues (Hua et al. 2022). This indicates that there may be a complex interplay between conserved regulatory genes and tissue-specific factors that affect the developmental differences of trichomes in different plant organs. Therefore, elucidating these spatiotemporal regulatory mechanisms is crucial for a comprehensive understanding of the complex processes involving trichome development across plant organs.

### The fruit trichome was a crucial regulator of post-harvest shelf life

The study of fruit trichomes was less explored compared to other plant parts, yet their impact on fruit appearance and post-harvest shelf life was significant. Given the distinct developmental pathways for trichome formation, which varied not only between species but also between different trichome types within the same species, deciphering the complex transcriptional regulation of trichome development is still challenging. Understanding the molecular regulation of fruit trichome development was essential to enhance fruit quality and extend shelf life. Tomatoes had an abundance of fruit trichomes that were easily broken off during handling. The exposure of fruit surface pores is a direct result of broken fruit trichomes. Therefore, the density of fruit trichomes in tomatoes affects postharvest water loss, offering new insights and technological routes for improving shelf life (Fich et al. 2020). Studies indicated that mutants with reduced cutin levels in tomato fruit do not always exhibit increased water loss, confirming the crucial role of trichome density in water retention (Bres et al. 2022; Isaacson et al. 2009). Breeding peaches for fewer or trichome-free varieties had the potential to reduce the allergy risks associated with trichomes, alter microbial communities, and extend the shelf life of the fruit (Cubells-Baeza et al. 2017; Shen et al. 2023).

### The interplay between trichome development and cuticle formation affects post-harvest shelf life

To understand fruit trichome functions, it is essential to explore their relationship with the cuticle. Both structures are important protective adaptations of the epidermis. The outer epidermal layer of plants is coated by the cuticle, a hydrophobic layer that provides an efficient barrier against water loss. In most of the fruits, the fruit surface is covered by a thicker cuticle than other tissues, and this cuticular membrane plays a crucial role in reducing water loss and providing resistance to pathogens and insects (Konishi et al. 2022; Wu et al. 2023b). Therefore, the abundance or composition of the cuticle, especially the cuticular wax content, significant determine water infiltration in most fruits during post-harvest (Macnee et al. 2021; Yang et al. 2022a). However, the integrity of the epidermal cells is a prerequisite for a significant positive correlation between cuticle thickness and water loss. For example, in leaves, water exchange occurs more frequently through stomata than through the cuticle layer. The primary reason why tomato fruit trichome density could influence post-harvest water loss is that fruit trichomes are easily broken during handling, leaving pores on the fruit surface the integrity of the fruit epidermis.

Both trichomes and cuticles are specialized structures of the epidermis and exhibit complex genetic interactions. Studies of many mutants have shown that defects in trichome formation can affect cuticle deposition and vice versa, in both leaf and fruit, suggesting that these processes are somehow linked. Several key transcription factors were already characterized as playing a role in both trichome and cuticle formation pathways, including in Arabidopsis (AtTCP14/AtTCP15, AtMYB106, AtMYB16) (Camoirano et al. 2020; Jakoby et al. 2008; Oshima et al. 2013), tomato (SlMX1, SlCD2, SlSHN3, SlMIXTA-like, SlWOOLLY) (Nadakuduti et al. 2012) (Ewas et al. 2016; Shi et al. 2013; Xiong et al. 2020) (Galdon-Armero et al. 2020), *Artemisia annua* (AaMIXITA1, AaHD8, AaTLR3) (Shi et al. 2018) (Dong et al. 2024; Yan et al. 2018), cucumber (CsMict, CsCER1) (Pan et al. 2021), peach (PpMYB25, PpMYB26) (Yang et al. 2022a), while likely many more still remain to be identified.

Overall, the synthesis of the regulatory mechanisms of trichome and cuticle development reveals common genetic pathways, underscoring the opportunity for targeted breeding strategies to improve agronomic traits that ensure better fruit quality, extended shelf life, and increased economic value. For example, a reduction in trichome density, a thinner and more digestible cuticle, and a long post-harvest shelf life are attractive traits for most of horticultural fruits if selection for palatability is to be achieved.

### Conclusions and perspectives

In conclusion, our review synthesizes current insights on the molecular regulation of fruit trichome development and their potential applications, underscoring the significance of these structures in postharvest water retention. The relationship between fruit trichome density and post-harvest shelf life offers new perspectives on extending fruit shelf life. Moreover, by elucidating the genetic interactions between trichome and cuticle formation, new opportunities for enhancing agronomic traits, thereby enhancing their economic viability and sustainability.

## Data Availability

Not applicable.

## Abbreviations

cd2/3: Cutin deficient 2/3
CK: Cytokinin
COI1: Coronatine-insensitive 1
csgl1: Cucumis sativus glabrous 1
csgl1/3: Cucumis sativus glabrous 1/3
CsCER1: Cucumber eceriferum 1
CHL: Cytokinin hydroxylase-like
EXPA: α-Expansin
fs1: Few spines 1
GA: Gibberellin
GA20ox1: Gibberellin 20-oxidases 1
HD-ZIP IV: Homeodomain-leucine zipper IV
HEC2: HECATE2
IAA: Indole-3-acetic acid
JA: Jasmonic acid
Ln: Lanata
LTR: Long terminal repeat
mGWAS: Metabolic genome-wide association study
mict: Micro-trichome
MYB: MYB transcription factor
NAC: NAM/ATAF/CUC
NAP1: Nucleosome assembly protein 1
NGS: Next-generation sequencing
Pru p 1/3: Peach lipid transfer protein 1/3
shn2: Shine2
TBH: Tiny branched hair
TCP: Teosinte branched1/Cycloidea/PCF
tril: Trichome-less
TS1: Tubercule size 1
TTG1: Transparent testa glabra 1
UV: Ultraviolet
WD-repeat: Tryptophan-Aspartic acid repeat
